# Dialectical Observation of Controllable Electrodeposited Ni Nanocones: the Unification of Local Disorder and Overall Order

**DOI:** 10.1186/s11671-020-03321-0

**Published:** 2020-04-22

**Authors:** Ruiqing Zou, Saidi Xiang, Jian Wang, Yuhe Li, Lin Gu, Yanyan Wang

**Affiliations:** 1 School of Materials Science and Engineering, Xihua University, Chengdu, 610039 People’s Republic of China; 2 School of Automotive Engineering, Chongqing University, Chongqing, 400044 People’s Republic of China; 3 School of Optoelectronic Science and Engineering & Collaborative Innovation Center of Suzhou Nano Science and Technology, Soochow University, Suzhou, 215006 People’s Republic of China

**Keywords:** Ni nanocones, Growth mechanism, Electrodeposition, Crystal modifier

## Abstract

Dense and ordered Ni nanocones with regular spiral textures had been successfully synthesized via a simple and inexpensive electrodeposition process in the solution containing sodium chloride (NaCl), nickel chloride hexahydrate (NiCl_2_·6H_2_O), and boric acid (H_3_BO_3_). After analyzing the microstructure, a more optimized possible growth mechanism of Ni nanocones was proposed, in which the growth process was divided into local and global aspects, named multi-dimensional growth mechanism of global order and local disorder. In an area small enough, any subtle state changes would cause disorder of Ni atom arrangement, which made the local microstructure appear disordered, but from a macro perspective, the difference between two adjacent disorders caused by different statuses was too small to be well reflected, only when the difference in state was large enough can the change be observed in the macroscopic appearance, so the global was orderly. Meanwhile, we found that the microstructure of Ni nanocones would be controlled in the electrodeposition solution by adjusting the experiment parameters such as the concentration of NaCl, NiCl_2_·6H_2_O, and H_3_BO_3_, which indirectly determined the microstructure in a large extent via controlling the generation of intermediate products and the pH.

## Introduction

Nanostructured metals with unique surfaces [1] were widely used in a variety of fields, such as surface modification [2], ultra-hydrophobic layers [3–5], supercapacitors [6], microelectronic interconnection [7], nanoprobes [8], solar cells [9], gas sensors [10, 11], catalysts [12–19], mechanical polishing slurries [20], diamond wheels [21], nanoscale precision surfaces [22, 23]. As a result, many preparation techniques of nanostructured metal surfaces had been proposed, including hydrothermal method [10, 11], sol-gel method [24], template method [25], chemical vapor deposition method [26], chemical reduction method [27], and microemulsion method [28]. However, these traditional methods required a great deal of cost and time [2, 29].

In order to overcome the defects of traditional preparation methods mentioned above, electrodeposition technology has attracted significant research interest and has experienced magnificent developments. It would achieve the target expectation even under the milder conditions for the electric field could increase the reaction rate [2]. The preparation of electrodeposited nanostructured metal surfaces did not require complex auxiliary equipment, which greatly reduced the cost and time. Therefore, a great deal of research had been done on the preparation technology and formation mechanism of nanostructured metal surface topography via electrodeposition [30].

In the specific electrodeposition preparation of nanostructured metal surface, the most representative method was the crystal modifier method [31]. The addition of crystal modifiers could affect the growth direction of crystals, so when reacted with an electrodeposition solution containing a specific crystal modifier, the metal nanostructured surface would grow in a specific direction. Therefore, the use of crystal modifier could easily obtain a specific, close-spaced, and regular 3D nanostructure on the surface of the metal substrate [32].

When a certain amount of ammonium chloride (NH_4_Cl), which was the most used crystal modifier, was added to Ni electrodeposition solution, the NH_4_^+^ would form complex ions with Ni^2+^ and cause the electrodeposited Ni crystal to grow along (111) crystal face. Therefore, by adding NH_4_Cl, it was easy to electrodeposit Ni nanocones that grow in a specific direction on the substrate metal surface and explain this phenomenon more accurately according to the growth mechanism of screw dislocation [33]. Furthermore, nickel, as a good ferromagnetic conductive metal, had the advantages of low price, wide use, and excellent corrosion resistance [34]. Ni nanocones obtained by electrodeposition with solution containing NH_4_Cl had important applications in gas-sensitive sensors [10, 11], ultra-hydrophobic surfaces [3–5], and catalysts [12–19].

In this work, we replaced NH_4_Cl with NaCl as the crystal modifier and prepared Ni nanocones successfully. Compared with NH_4_Cl, NaCl was non-toxic, gentle, and stable. In addition, we put forward the possible specific complex structure and its role in the process of electrodeposition by combining hybrid orbital theory, molecular orbit theory, and actual characterization results. The growth mechanism of Ni nanocones electrodeposited in the solution containing NaCl, which was quite different from common screw dislocation-driven crystal growth mechanism [35], was expounded, and the effects of electrodeposition time and the concentration of NaCl, NiCl_2,_ and H_3_BO_3_ on the nanostructure of Ni nanocones were analyzed. Through the analysis of factors affected by the nanostructure of Ni nanocones, the controlled preparation was preliminarily realized, which would be instructive for the preparation of other special-shaped Ni nanocones in the future.

## Materials and Methods

### Materials

All chemical reagents were analytical pure and could be used directly for chemical reactions. The length, width, and thickness of Ni tablets (cathode and anode) used in our experiment were 70 mm, 25 mm, and 0.08 mm, respectively. Sodium chloride (NaCl), nickel chloride hexahydrate (NiCl_2_·6H_2_O), boric acid (H_3_BO_3_), hydrochloric acid (HCl), and anhydrous ethanol (CH_3_CH_2_OH) were purchased from ChengDu Chron Chemicals Co., Ltd., China.

### Surface Treatment and Sample Preparation

In a standard process, two Ni tablets (cathode and anode) were ultrasonically cleaned in deionized water and ethanol, respectively. The cathode Ni tablet was roughened in HCl (25 wt.%, 60 °C) for 30 min. Subsequently, Ni nanocones were electrodeposited on the as-prepared Ni tablets (cathode) from an aqueous solution containing NiCl_2_·6H_2_O (200 g/L), NaCl (100 g/L), and H_3_BO_3_ (50 g/L). The temperature (60 °C), current density (0.1 A), and electrodeposition time (20 min) should be regulated and another Ni tablet was employed as the anode to provide Ni ions (Ni^2+^). After the electrodeposition, the cathode was ultrasonically cleaned in deionized water and then ethanol and finally dried in oven for 30 min, respectively. For further comparison, the electrodeposition time was controlled from 5 min to 50 min, and the concentration of NaCl, NiCl_2_·6H_2_O, and H_3_BO_3_ was changed from 0 to 167 g/L, 0 to 400 g/L, and 0 to 50 g/L, respectively (Table 1).

**Table 1:** Values of NaCl, NiCl_2_·6H_2_O, and H_3_BO_3_ concentration and electrodeposition time of different samples

Sample	Electrodeposition time (min)	NaCl concentration (g/L)	NiCl_2_·6H_2_O concentration (g/L)	H_3_BO_3_ concentration (g/L)
Ni_355_	5	100	200	50
Ni_370_	20	100	200	50
Ni_400_	50	100	200	50
Ni_270_	20	0	200	50
Ni_437_	20	167	200	50
Ni_170_	20	100	0	50
Ni_570_	20	100	400	50
Ni_320_	20	100	200	0
Ni_345_	20	100	200	25

### Characterization

The scanning electron microscope (SEM) images and corresponding energy dispersive spectroscopy (EDS) were obtained through FEI Inspect F50 (Thermo Fisher, USA) operating at 20 kV. The X-Ray diffraction (XRD) patterns were measured using a D8 advance (BRUKER, Germany) X-ray diffractometer with a Cu Kα radiation (*λ* = 1.5406 Å). The Fourier Transform infrared spectroscopy (FTIR) pattern was measured using a Nicolet iS 10 (Thermo Fisher, USA) with an ATR module.

## Results and Discussion

### Determination of Intermediate Products

**Fig. 1:**
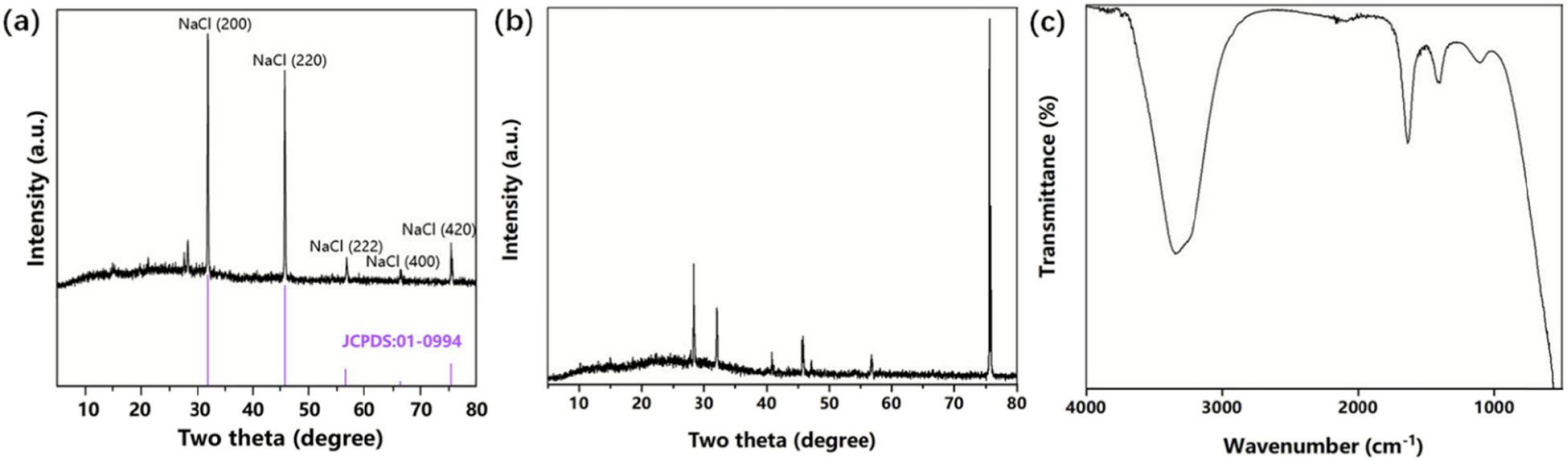
The XRD patterns of electrodeposition solution which were heated and dried with an alcohol lamp (**a**) and with an oven (60 °C) (**b**). The FTIR pattern of electrodeposition solution (**c**) after electrodeposition

Usually, when NH_4_Cl was used as crystal modifier, the NH_4_^+^ would form complex ions with the Ni^2+^ during the electrodeposition process [36]. Therefore, when NaCl was used as a crystal modifier, the solution might produce complex ions, which could promote the conduct of electrodeposition. Figure 1 shows the XRD patterns of electrodeposition solution which were heated and dried with an alcohol lamp (Fig. 1a) and with an oven (60 °C) (Fig. 1b), respectively, and the FTIR pattern of electrodeposition solution (Fig. 1c) after electrodeposition. We could see five different peaks clearly in Fig. 1a, which were NaCl (111), (200), (220), (222), and (400), respectively, compared with XRD standard PDF card. This indicated that the chemical bonds of the target product had been broken after the electrodeposition solution treated at high temperatures (alcohol lamp), in other words, the target product was resistant to poor high-temperature performance. Afterwards, we heated and dried the electrodeposition solution at a lower temperature (60 °C, oven), and the resulting XRD pattern was shown in Fig. 1b. Unfortunately, compared to the XRD standard PDF cards for all possible compounds, nothing would correspond to these peaks. This suggested that the resulting target product was not a common general compound and might be a rare and special complex. Figure 1c shows the FTIR pattern of electrodeposition solution after electrodeposition, where we could find a peak around 1500 cm^−1^, which was consistent with the characteristic peak (1499 cm^−1^) that the ionic liquid containing Ni halide anion had [37]. Therefore, combined with XRD and FTIR patterns, we considered that some complex ions ([Ni_x_Cl_y_]^z−^), which still presented after the reaction and had poor high-temperature resistance, were generated in the solution during the electrodeposition process.

When Cl^−^ encountered Ni^2+^ in the solution, Ni^2+^ would be hybridized according to the hybrid orbital theory and form [Ni(H_2_O)_2_]Cl_4_. According to Fernandes et al., when the temperature was above 30 °C, the water molecules would be replaced by Cl^−^. According to molecular orbital theory, each lone pair of Cl^−^ did not occupy a single orbit but divided all four orbits equally, the transition from an octahedral complex to a tetrahedral complex occurred [38]. Thus, each lone pair was consistent in both number and energy of occupied orbits, and in theory, the resulting [NiCl_4_]^2−^ presented a structure of regular tetrahedron in space.

### Effect of Electrodeposition Time and Crystal Modifier

**Fig. 2:**
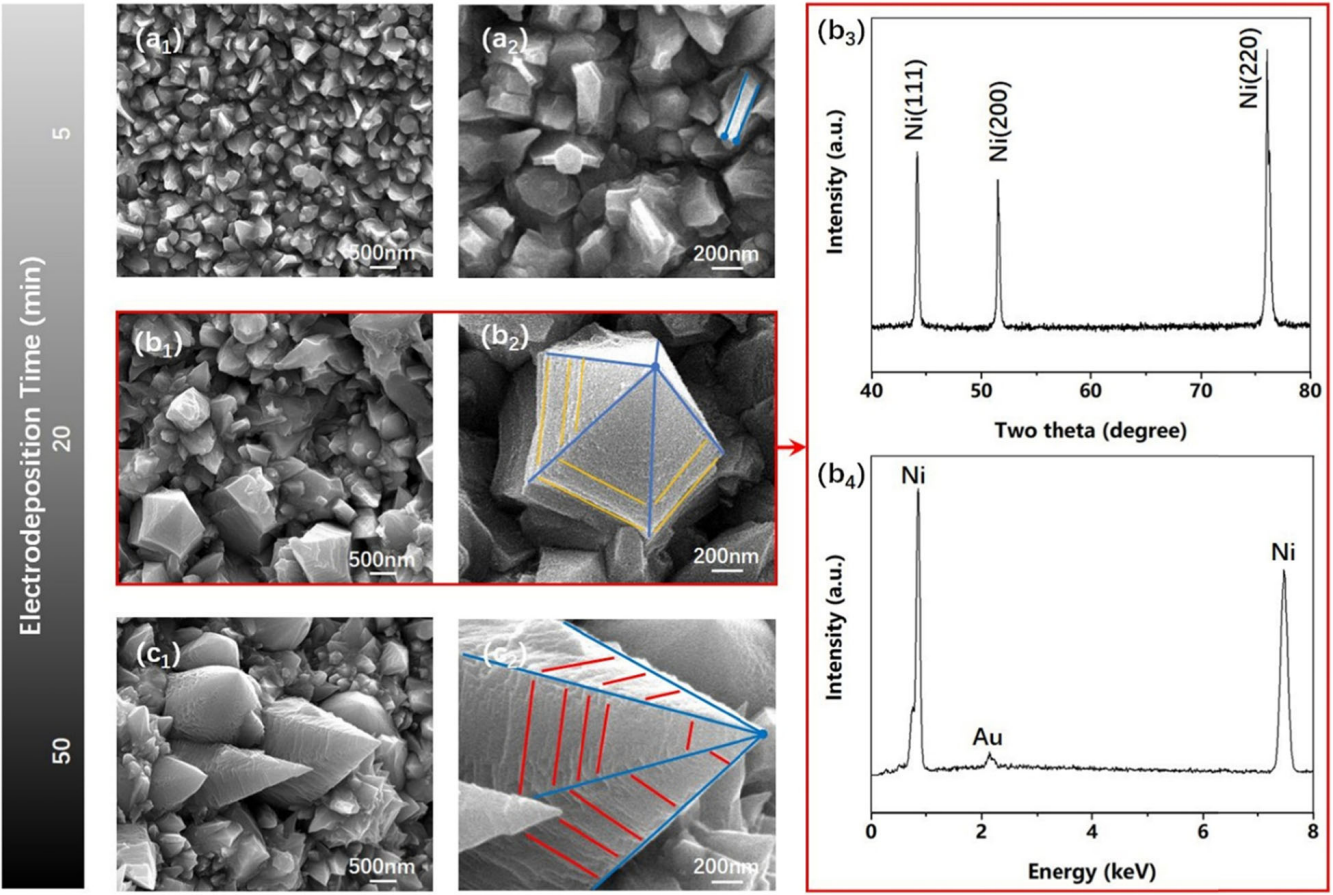
Low-magnification SEM images on the surface morphology of Ni nanocones with different electrodeposition time (**a**_**1**_, **b**_**1**_, **c**_**1**_) and their high-magnification SEM images (**a**_**2**_, **b**_**2**_, **c**_**2**_), respectively. XRD pattern (**b**_**3**_) and EDS pattern (**b**_**4**_) of Ni_370_ nanocones

Figure 2 shows low (Fig. 2 a_1_–c_1_) and high (Fig. 2a_2_–c_2_) magnification SEM images of Ni_355_/Ni_370_/Ni_400_ nanocones with different electrodeposition time (5 min, 20 min, 50 min) , XRD and EDS (Fig. 2 b_3_, b_4_) patterns of typical Ni_370_ nanocones (Fig. 2b_1_), respectively. It was clear from SEM images that the cathode surface was covered by small and dense plate-like structure through a short electrodeposition time (5 min), and nanocone structures were gradually formed with the increase of electrodeposition time (20 min). With nanocones grown further, the sharp corners were clearer and more textures for longer periods of electrodeposition time (50 min). It was clear from the XRD pattern that there were three different diffraction peaks and all of them were consistent with pure Ni phase with face center cubic (fcc) structure, and no other impurity peaks such as NiO or Ni(OH)_2_ could be detected. Meanwhile, it was obvious that Ni mainly grew along (220) crystal face. As can be seen from the EDS pattern of typical Ni_370_ nanocones, only Au and Ni could be confirmed, indicating that what electrodeposited on the cathode was pure Ni_370_ nanocones without any contamination (Ni was a magnetic material, in order to attenuate magnetically and protect the probe, it needed to be sprayed with gold during SEM characterization).

**Fig. 3:**
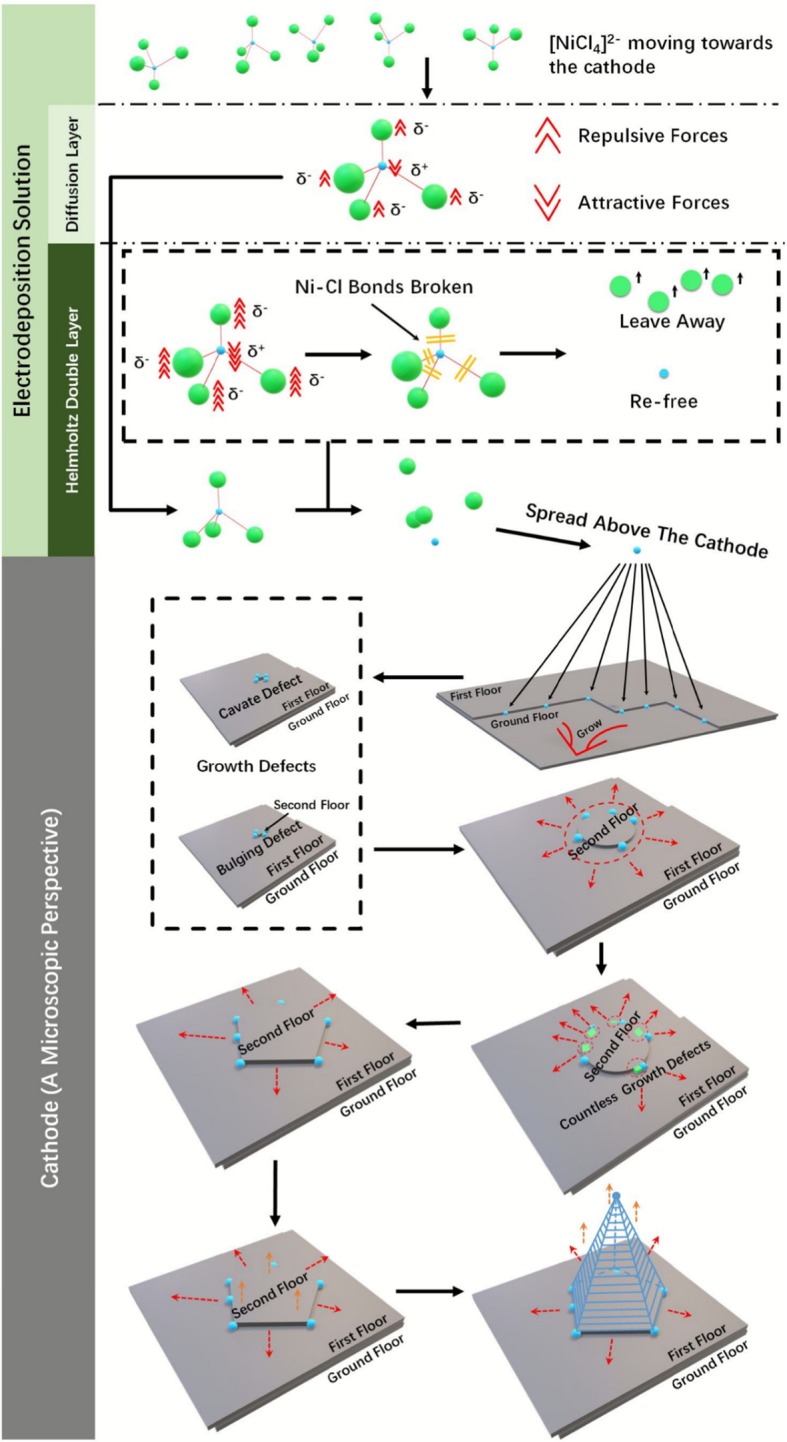
Multi-dimensional growth mechanism of global order and local disorder: the fracture of [NiCl_4_]^2−^ coordinate covalent bonds, the motion state, and assembly mode of Ni^2+^ on the cathode

Figure 3 shows the specific growth mechanism of Ni_370_ nanocones, named multi-dimensional growth mechanism of global order and local disorder. At the beginning, [NiCl_4_]^2−^ in the solution moved towards cathode under the action of electric field, Cl^−^ with negative electricality in the [NiCl_4_]^2−^ produced repulsive force against electrons on the cathode, whereas Ni^2+^ with positive electricality were attracted to the cathode after entering the diffusion layer. Both repulsive and attractive forces increased significantly, after entering the Helmholtz double layer, the coordinate covalent bonds (Ni–Cl) of [NiCl_4_]^2−^ were broken, and then the re-free Cl^−^ (Cl in the broken Ni–Cl bonds) moved against the cathode while the re-free Ni^2+^ (Ni in the broken Ni–Cl bonds) moved towards the cathode. Re-free Ni^2+^ moved in parallel over the cathode and chose the easiest place to attach, usually at the defects and at the steps, for the electrodeposition growth of Ni. Defects (normally, cavate defects and bulging defects) would inevitably occur during the 2D growth of Ni on the cathode, transformed the growth from 2D to 3D (the effects of defects on flat growth was not considered here, but considered the effects on the *z*-axis direction growth). Ideally, the chances of a new layer growing in each direction caused by a single defect were the same, in other words, the new layer should grow outward in a circular shape (the effects of defects on *z*-axis direction growth was not considered here, but considered the effects on the flat growth). However, what we saw from Fig. 2 b_2_ and c_2_ were pyramids rather than circular cones, because the growth of a new layer was still accompanied by a large number of defects that appeared at the frontiers of growth; each defect would make its state differ slightly from the surrounding growth frontiers (a very small range); thus, the resulted Ni nanocone was strictly an *N*-sided polygonal pyramid, which was called local disorder. Although there were different statuses (growth rate, growth direction, etc.) between two adjacent defects, it was negligible compared to those two defect aggregation points that were far apart. At the macro level, only two defect aggregation points with sufficient status differences that deserved attention and isolated defects within certain statuses ranges were “merged,” Ni nanocones we observed shown a pyramid (triangular, quadrangular, pentagonal, hexagonal pyramid, etc.), which was called global order (Fig. S1).

### Effect of Components

In order to further study the specific effect of components in the solution, control variates were used to make preliminary analysis of the surface nanostructure obtained by electrodeposition at different concentrations of NaCl, NiCl_2_, and H_3_BO_3_, respectively, and draw corresponding conclusions.

### Effect of NaCl

**Fig. 4:**
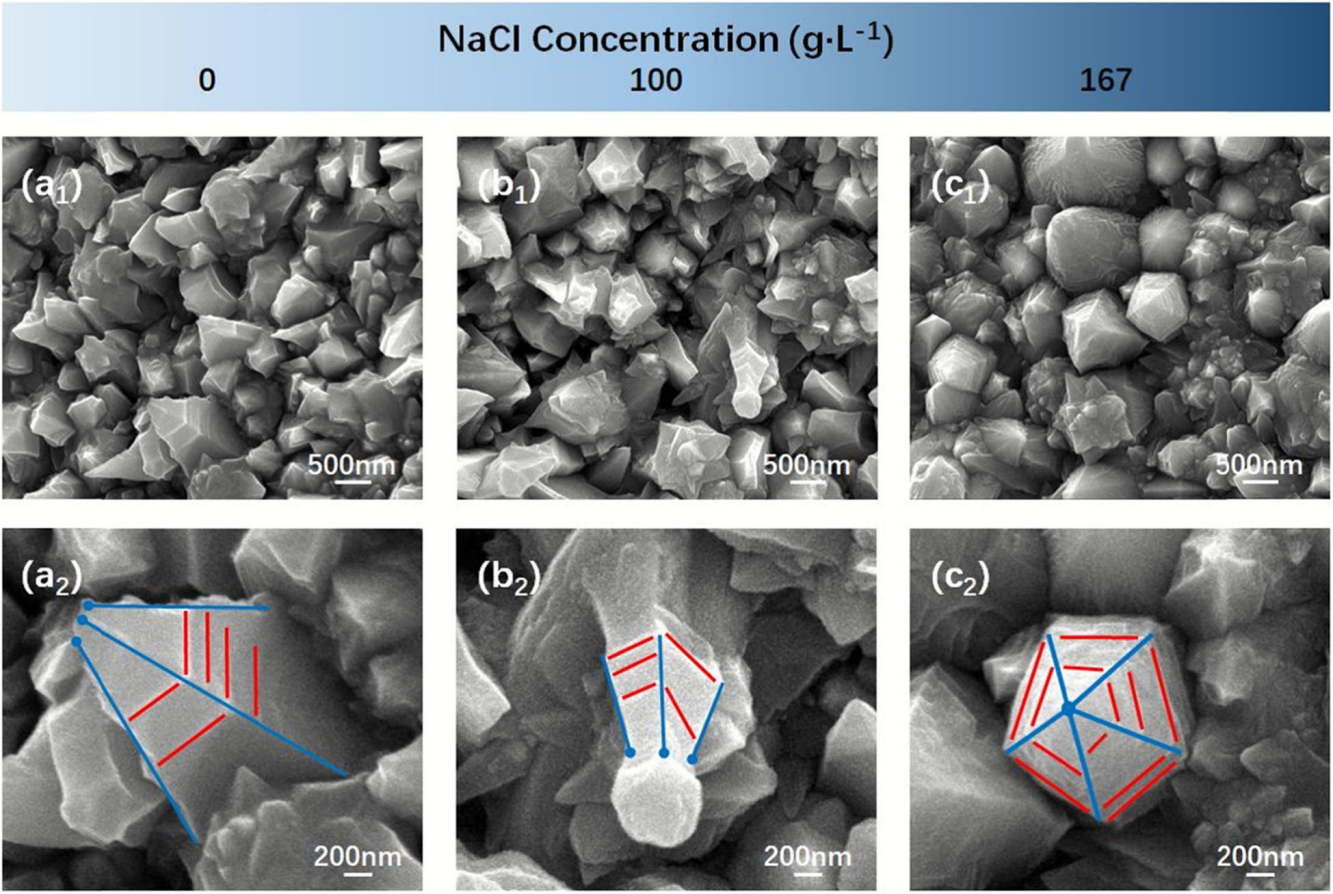
Low- and high-magnification SEM images of cathode that electrodeposited at different NaCl concentrations: 0 g/L (**a**_**1**_, **a**_**2**_), 100 g/L (**b**_**1**_, **b**_**2**_), and 167 g/L (**c**_**1**_, **c**_**2**_), respectively

Figure 4 shows the low- and high-magnification SEM images of cathode that electrodeposited under different NaCl concentrations at 0 g/L (Fig. 4 a_1_ and a_2_), 100 g/L (Fig. 4 b_1_ and b_2_) and 167 g/L (Fig. 4c_1_ and c_2_), respectively. When NaCl was not added to the solution, the cathode surface was covered by blocky Ni_270_ nanostructure (Fig. 4 a_1_), and although some blocks had a spire-shaped tendency at the apex (Fig. 4a_2_), it seemed to be just called undeveloped Ni_270_ nanocones. The reason why it resulted in a large number of undeveloped Ni nanocones on the surface of the cathode was that only NiCl_2_ in the solution provided Cl^−^, which made too less Cl^−^ to produce a large number of [NiCl_4_]^2−^, and further seriously hindered the generation of Ni_270_ nanocones. When the concentration of NaCl increased (100 g/L), it could be observed that there were still some undeveloped Ni_370_ nanocones (Fig. 4 b_1_), but a more pronounced trend of Ni nanocones in some places (Fig. 4b_2_). This was because the addition of NaCl in the solution greatly alleviated the lack of Cl^−^, promoted the formation of [NiCl_4_]^2−^, but it still could not reach a ratio of 1:4 (n(Ni^2+^):n(Cl^−^)), and the cathode surface was covered by many undeveloped Ni_370_ nanocones. Continuing to increase the concentration of NaCl to 167 g/L, it could be found that most of the cathode surface was covered by Ni_437_ nanocones which made it almost impossible to detect undeveloped Ni_437_ nanocones (Fig. 4 c_1_, c_2_). The large amount of Cl^−^ in the solution made it possible to produce huge number of [NiCl_4_]^2−^, which greatly promoted the generation of electrodeposited Ni_437_ nanocones.

### Effect of NiCl_2_

**Fig. 5:**
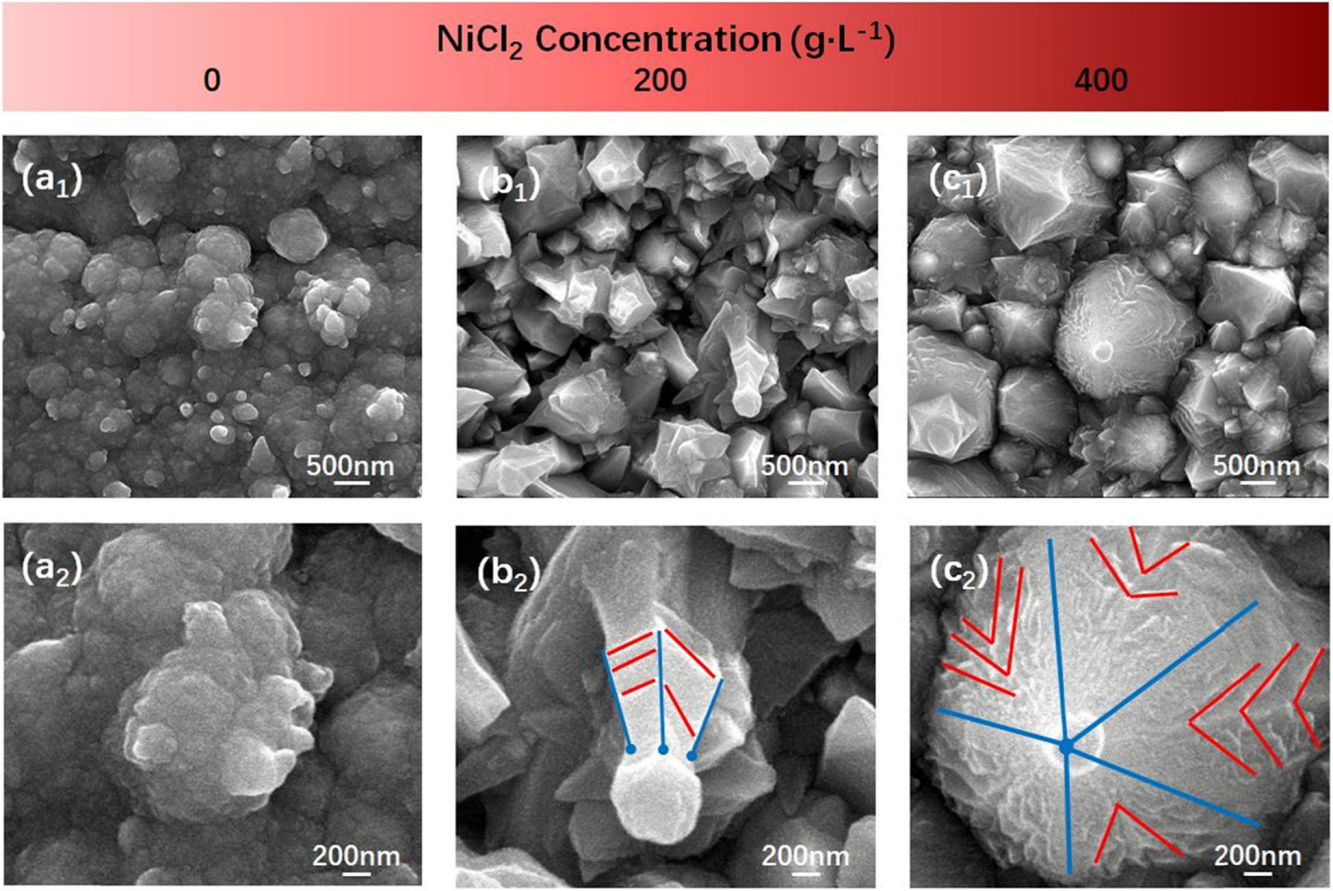
Low- and high-magnification SEM images of cathode that electrodeposited at different NiCl_2_ concentrations: 0 g/L (**a**_**1**_, **a**_**2**_), 200 g/L (**b**_**1**_, **b**_**2**_), and 400 g/L (**c**_**1**_, **c**_**2**_), respectively

Figure 5 shows the low- and high-magnification SEM images of cathode that electrodeposited under different NiCl_2_ concentrations: 0 g/L (Fig. 5 a_1_ and a_2_), 200 g/L (Fig. 5 b_1_ and b_2_), 400 g/L (Fig. 5 c_1_ and c_2_), respectively. It was clear from Fig. 5 a_1_ that the cathode surface was covered by a cotton floc-shaped structure, and the entire surface structure tended to be more densely stacked with Ni balls but no cones structure (Fig. 5a_2_). The reason why Ni^2+^ electrodeposited on the cathode in a slow and more average way and caused a cotton floc-shaped structure was that there was no Ni^2+^ in the solution before electrodeposition; Ni^2+^ required for electrodeposition all came from those Ni atoms which lost electrons on the anode, resulting in low concentration of Ni^2+^ in the solution, so even if there were a large number of Cl^−^, the generation of [NiCl_4_]^2−^ was rare, which seriously hindered the formation of Ni nanocones structure. After increasing the concentration of NiCl_2_ in the solution to 200 g/L, the electrodeposited cathode surface was covered by some Ni_370_ nanocones and others undeveloped (Fig. 5 b_1_), the entire surface was rough and fragmented (Fig. 5b_2_). NiCl_2_ added to the solution greatly increased the generation of [NiCl_4_]^2−^, prompted the formation of Ni_370_ nanocones, but a part of underdeveloped Ni_370_ nanocones indicated that the concentration might not have reached the optimal level. When the concentration of NiCl_2_ reached 400 g/L, the cathode surface was covered by a large number of huge Ni_570_ nanocones (Fig. 5 c_1_), some of them presented vaguely visible edges but more conical shapes, and the cone surface was full of texture, with sharp angles and tips pointed to the cone vertex (Fig. 5 c_2_, red lines). Theoretically, the concentration of Ni^2+^ provided by NiCl_2_ (400 g/L) was far greater than that desired, which instead highlighted the lack of Cl^−^; then, a large number of Ni^2+^ electrodeposited on the cathode during a short period of time resulted in Ni_570_ nanocones grown too fast to present the local disorder characteristics but emerged cone structure.

### Effect of H_3_BO_3_

In the series of experiments, H_3_BO_3_ was the role of pH regulator, since boron (B) was an electron-deficient atom, it could combine with hydrogen oxygen root ions (OH^−^) from the water molecules, and thereby release hydrogen ions (H^+^) (Eq. 1).

Eq. 1 $$ \mathrm{B}{\left(\mathrm{OH}\right)}_3+{\mathrm{H}}_2\mathrm{O}\to \mathrm{B}{\left(\mathrm{OH}\right)}_4^{-}+{\mathrm{H}}^{+} $$

**Fig. 6:**
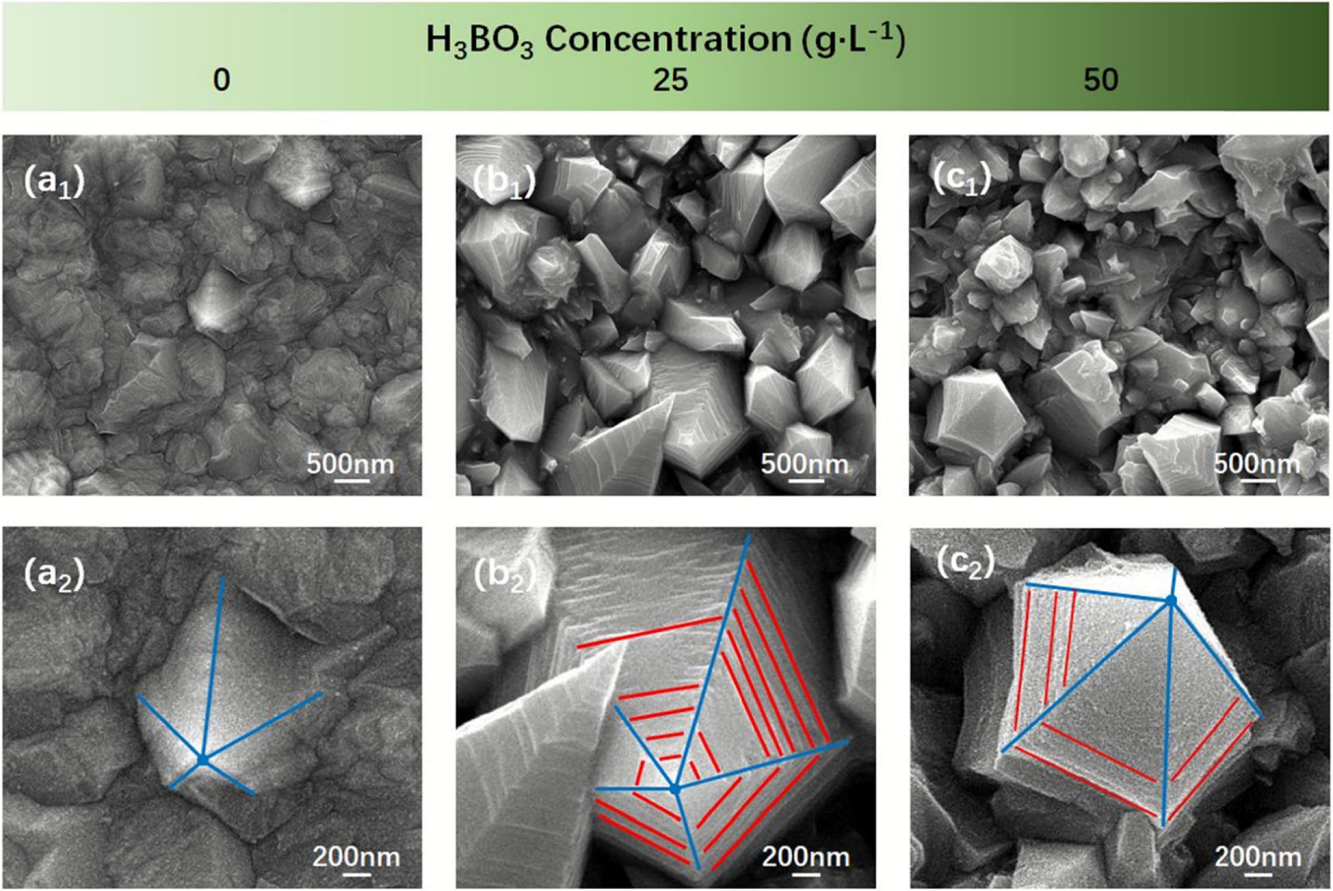
Low- and high-magnification SEM images of cathode that electrodeposited at different H_3_BO_3_ concentrations: 0 g/L (**a**_**1**_, **a**_**2**_), 25 g/L (**b**_**1**_, **b**_**2**_), and 50 g/L (**c**_**1**_, **c**_**2**_), respectively

Figure 6 shows the low- and high-magnification SEM images of cathode that electrodeposited from different H_3_BO_3_ concentrations: 0 g/L (Fig. 6 a_1_ and a_2_), 25 g/L (Fig. 6 b_1_ and b_2_), 50 g/L ((Fig. 6c_1_ and c_2_), respectively. In Fig. 6a_1_, it could be clearly seen that the cathode was covered with a relatively flat electrodeposition layer, and some areas had slight protrusions (Fig. 6 a_2_), but no Ni_320_ nanocones structure overall. When there was no H_3_BO_3_ in the solution, only electrolyzed water reaction would occur near the cathode, so the solution was generally in an acid-base equilibrium state, and Ni^2+^ were almost immune to the influence of OH^−^ or H^+^, resulting in a flat electrodeposition layer. Adding H_3_BO_3_ to 25 g/L in the solution, we could observe obvious huge Ni_345_ nanocones structure from Fig. 6 b_1_, while the texture trend was complex, the edges and corners were clear (Fig. 6b_2_). This was due to the addition of H_3_BO_3_, which supplied more H^+^ near the cathode, resulting in a weak acidity environment. When the concentration of H_3_BO_3_ in the solution reached 50 g/L, the size of Ni_370_ nanocone was reduced, compared to Ni_345_ nanocones, but the surface texture was smoother. This was due to the large number of H_3_BO_3_, which made the solution appear weak acidic (slightly more acidic than the previous one), the excessive H^+^ affected the size of Ni_370_ nanocones but made the surface more regular.

Overall, the size of Ni nanocones was not proportional relationship to the amount of H_3_BO_3_ in the solution. This might be that when the solution was free of H_3_BO_3_, the negatively charged OH^−^ interfered with the positive electricality Ni^2+^, which affected the motion of Ni^2+^. On another hand, when a large amount of H_3_BO_3_ was presented in the solution, a lot of H^+^ would attach the cathode surface to generate hydrogen (H_2_), but due to the small size of H^+^ itself, the influence on the nanostructure of Ni nanocones was quite limited.

## Conclusion

In summary, we had successfully synthesized Ni nanocones via electrodeposition process in the solution containing NiCl_2_, NaCl, and H_3_BO_3_. The results have shown that the intermediate product was a special complex, which still presented after the reaction and had poor high-temperature resistance, and that Ni nanocones were pure Ni with fcc structure, grown mainly along (220) crystal face. Moreover, the specific structure of the intermediate product was supported by known mature theoretical systems, the nanostructure and electrodeposition process of the products were investigated, and the probable formation mechanism of Ni nanocones was discussed based on the experimental results. Meanwhile, we found that the nanostructure of Ni nanocones could be controlled by adjusting the experimental conditions such as the concentration of NiCl_2_, NaCl, and H_3_BO_3_, respectively. Therefore, establishing appropriate parameters was key point for the synthesis of Ni crystals with nanocones structure via this electrodeposition approach. Additionally, we expected that this novel strategy could be possibly extended to some other magnetic metals to synthesize controllable nanocone structure.

## Supplementary information

**Additional file 1:.Figure S1.** The supplementary of multi-dimensional growth mechanism of global order and local disorder

## Data Availability

The datasets used for supporting the conclusion are included in the article and the supporting file.
