# Supplementary material for: Dialectical Observation of Controllable Electrodeposited Ni Nanocones: the Unification of Local Disorder and Overall Order
Source: Nanoscale Res Lett. 2020 Apr 22;15:91. doi: 10.1186/s11671-020-03321-0 (PMC7176806; doi:10.1186/s11671-020-03321-0)
Supplement: Supplementary file 1 — Additional file 1:.Figure S1. The supplementary of multi-dimensional growth mechanism of global order and local disorder [file 11671_2020_3321_MOESM1_ESM.docx]

***Electronic Supplementary Information***

***For***

**Dialectical observation of controllable electrodeposited Ni nano cones: the unification of local disorder and overall order**

Ruiqing Zou^1^, Saidi Xiang^2^, Jian Wang^1*^, Yuhe Li^1^, Lin Gu^1*^ and Yanyan Wang^3^

^1^ School of Materials Science and Engineering, Xihua University, Chengdu 610039, People's Republic of China. E-mail: wangjianxhu@163.com

^2^ School of Automotive Engineering, Chongqing University, Chongqing 400044, People's Republic of China.

^3^ School of Optoelectronic Science and Engineering & Collaborative Innovation Center of Suzhou Nano Science and Technology, Soochow University, Suzhou 215006, People’s Republic of China.





Fig. S1 The supplementary of multi-dimensional growth mechanism of global order and local disorder

Fig. S1 shows a supplementary explanation of multi-dimensional growth mechanism of global order and local disorder. At a small magnification, the surface of a single Ni nano cone was regular and smooth (Fig. S1a), but when it was enlarged, the surface became rough and irregular (Fig. S1b). This was because the smallest repeatable unit of Ni nano growth frontier was Ni cell, cube structure, which made the growth frontier of Ni nano cone be jagged at sufficient magnification (growth on a single floor) (Fig. S1c). In an ideal state, each layer of growth expanded evenly outwards from the center of the circle, forming a circle, which superimposed and presented a perfect conical shape (Fig. S1d). However, there were many defects among the growth frontier, and the distribution of these defects was extremely uneven, which allowed defect aggregation points existing at the forefront of growth and changed the shape of this layer from circular to polygon. At the same time, these defect aggregation points also affected the location of defect aggregation point sited on the next layer, which made each location of defect aggregation point on the subsequent layer substantially same and eventually formed a pyramid (Fig. S1e).
